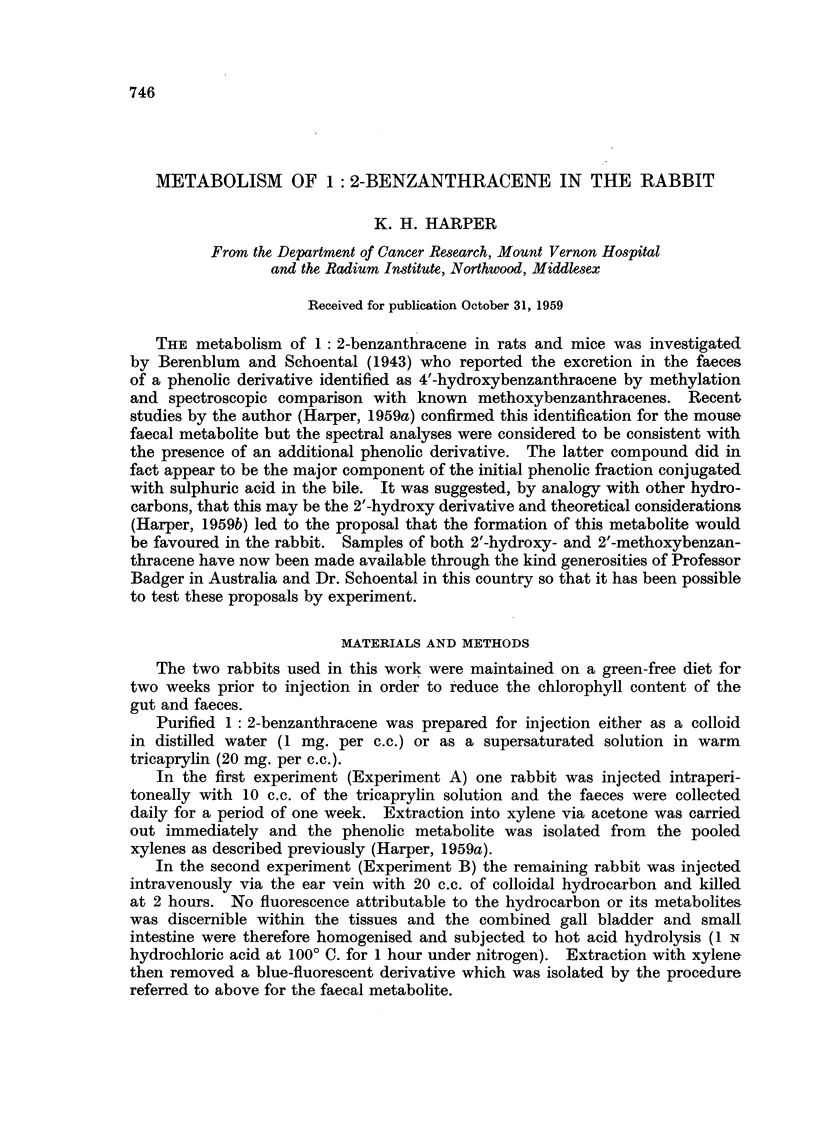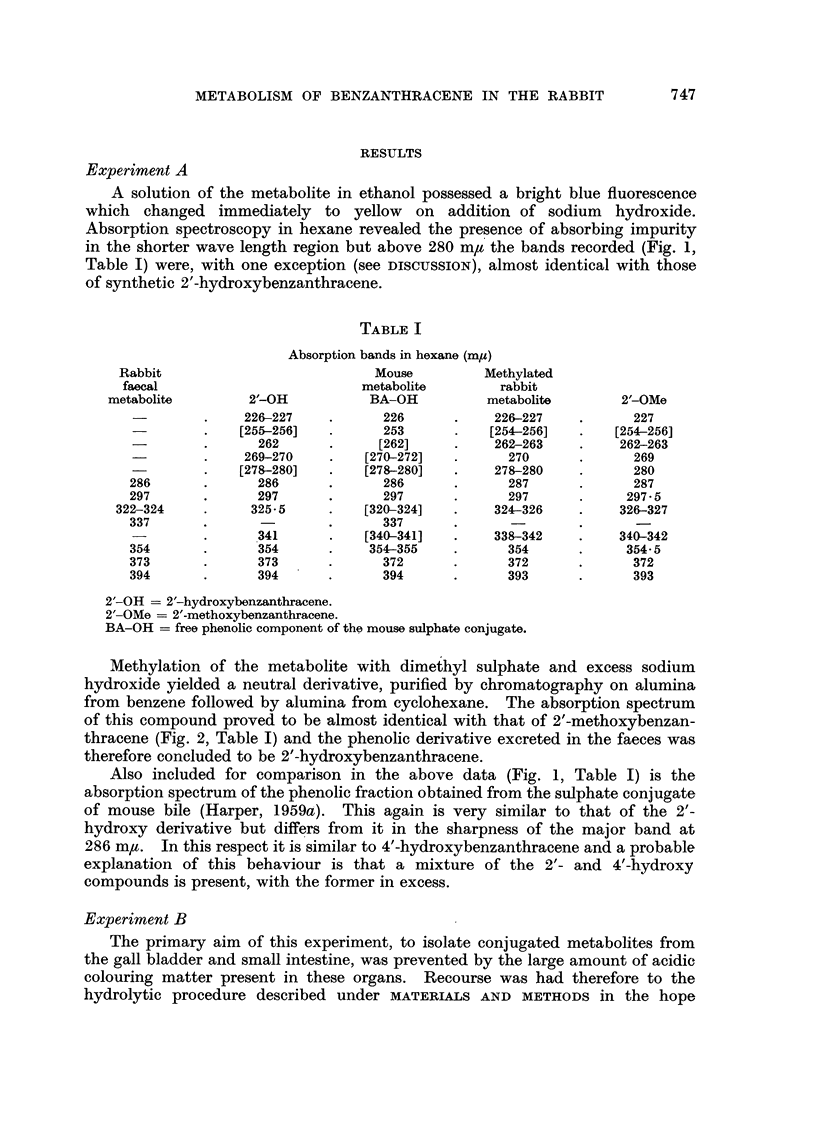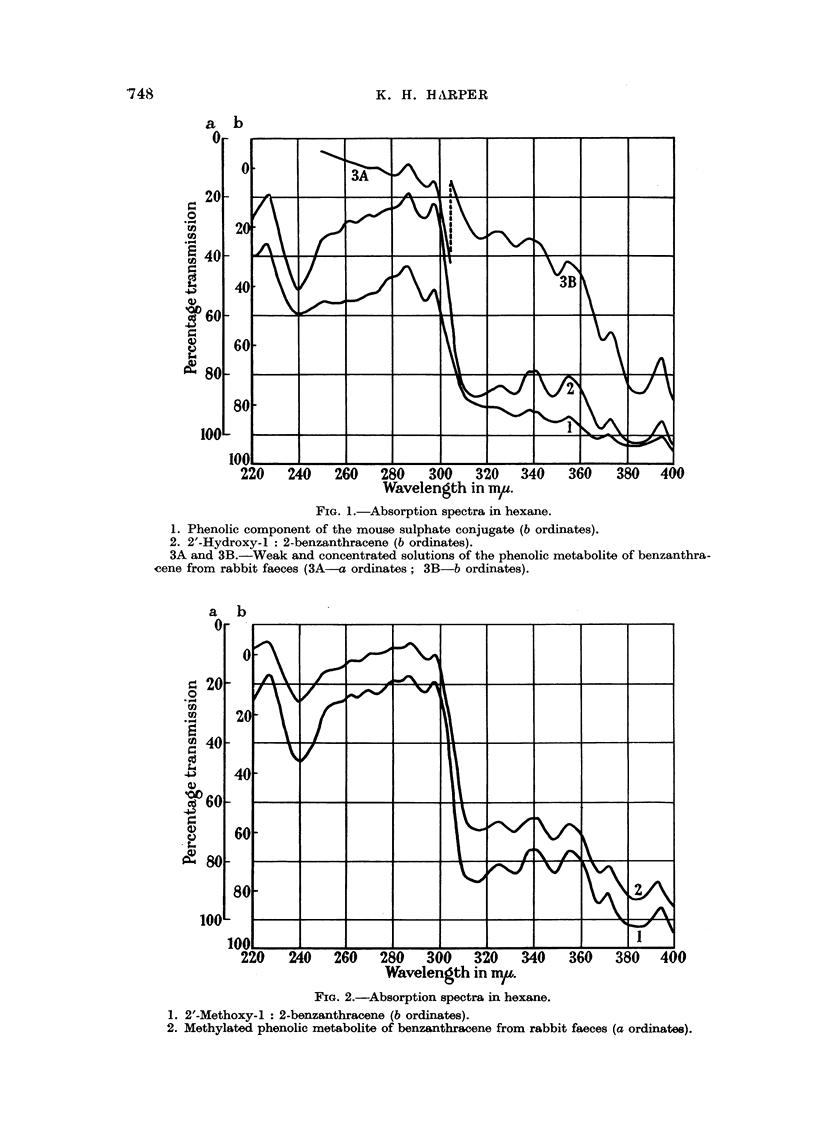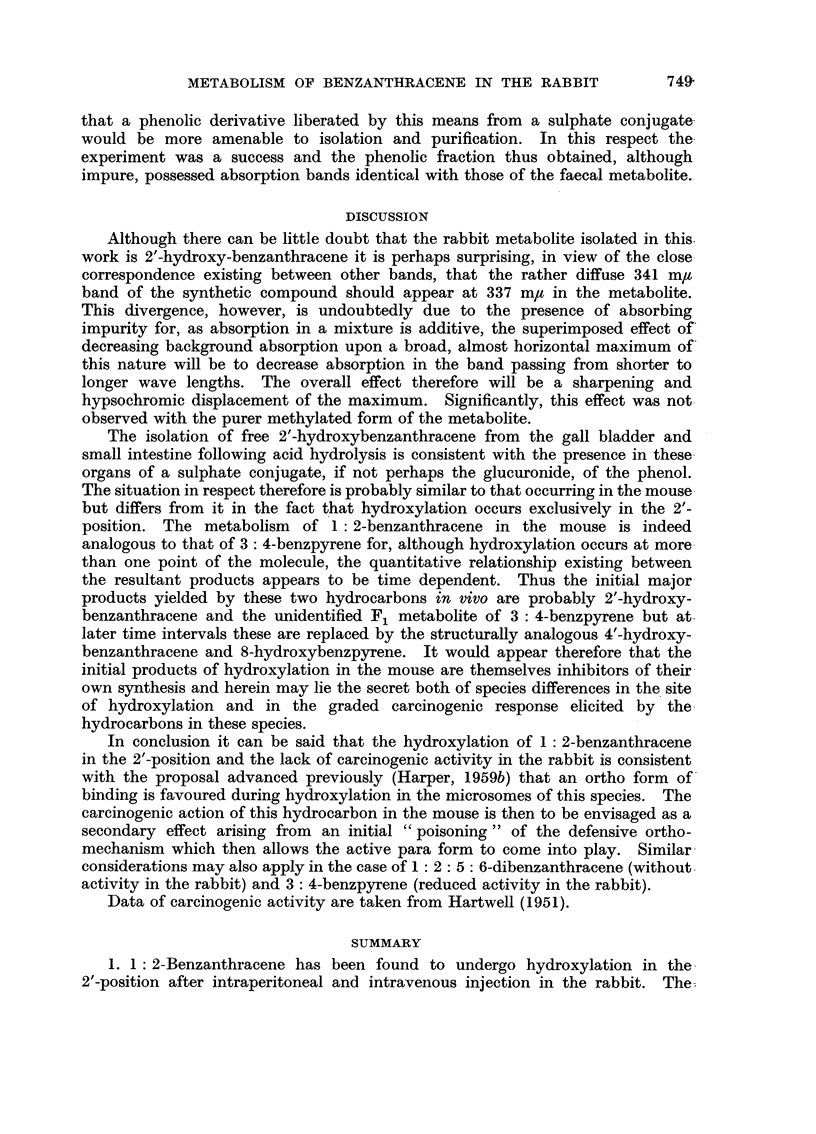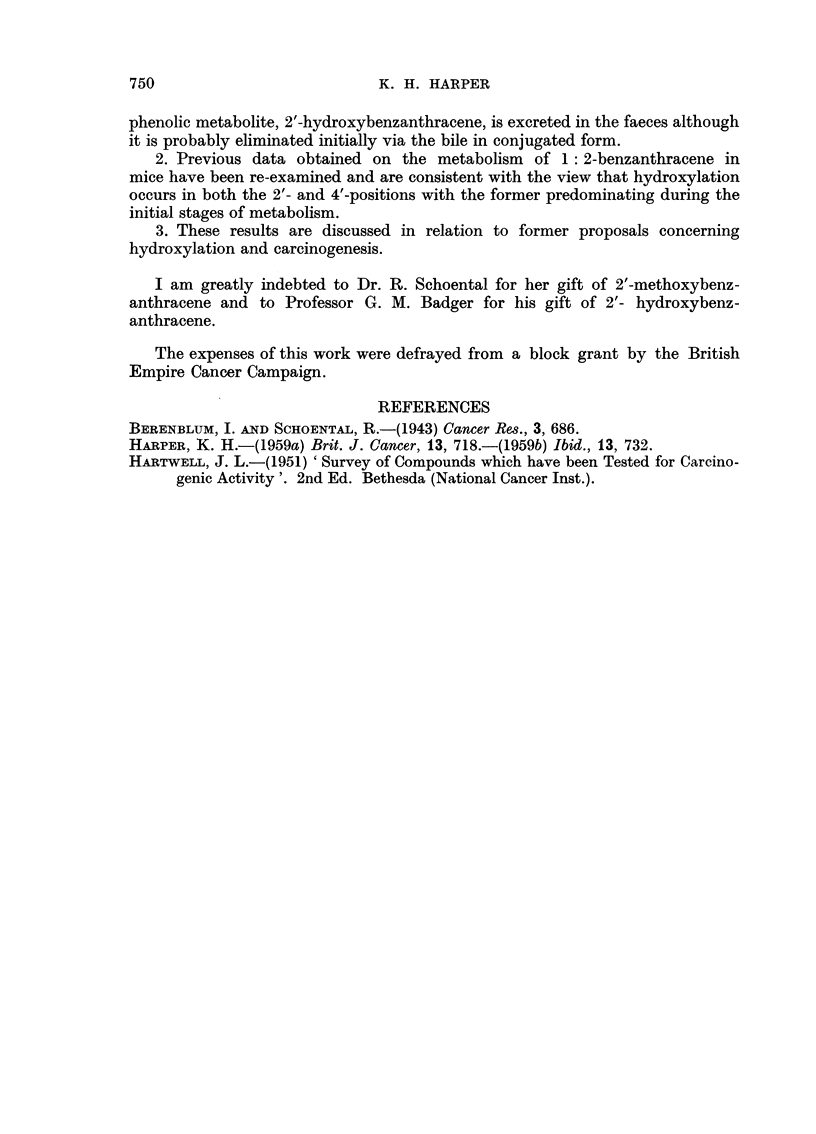# Metabolism of 1:2-Benzanthracene in the Rabbit

**DOI:** 10.1038/bjc.1959.83

**Published:** 1959-12

**Authors:** K. H. Harper


### METABOLISM OF 1 : 2-BENZANTHRACENE IN THE RABBIT

K. H. HARPER

*From the Department of Cancer Research, Mount Vernon Hospital and the Radium Institute, Northwood, Middlesex*

Received for publication October 31, 1959

THE metabolism of 1 : 2-benzanthracene in rats and mice was investigated by Berenblum and Schoental (1943) who reported the excretion in the faeces of a phenolic derivative identified as 4′-hydroxybenzanthracene by methylation and spectroscopic comparison with known methoxybenzanthracenes. Recent studies by the author (Harper, 1959*a*) confirmed this identification for the mouse faecal metabolite but the spectral analyses were considered to be consistent with the presence of an additional phenolic derivative. The latter compound did in fact appear to be the major component of the initial phenolic fraction conjugated with sulphuric acid in the bile. It was suggested, by analogy with other hydrocarbons, that this may be the 2′-hydroxy derivative and theoretical considerations (Harper, 1959*b*) led to the proposal that the formation of this metabolite would be favoured in the rabbit. Samples of both 2′-hydroxy- and 2′-methoxybenzanthracene have now been made available through the kind generosities of Professor Badger in Australia and Dr. Schoental in this country so that it has been possible to test these proposals by experiment.

### MATERIALS AND METHODS

The two rabbits used in this work were maintained on a green-free diet for two weeks prior to injection in order to reduce the chlorophyll content of the gut and faeces.

Purified 1 : 2-benzanthracene was prepared for injection either as a colloid in distilled water (1 mg. per c.c.) or as a supersaturated solution in warm tricaprylin (20 mg. per c.c.).

In the first experiment (Experiment A) one rabbit was injected intraperitoneally with 10 c.c. of the tricaprylin solution and the faeces were collected daily for a period of one week. Extraction into xylene via acetone was carried out immediately and the phenolic metabolite was isolated from the pooled xylenes as described previously (Harper, 1959*a*).

In the second experiment (Experiment B) the remaining rabbit was injected intravenously via the ear vein with 20 c.c. of colloidal hydrocarbon and killed at 2 hours. No fluorescence attributable to the hydrocarbon or its metabolites was discernible within the tissues and the combined gall bladder and small intestine were therefore homogenised and subjected to hot acid hydrolysis (1 N hydrochloric acid at 100° C. for 1 hour under nitrogen). Extraction with xylene then removed a blue-fluorescent derivative which was isolated by the procedure referred to above for the faecal metabolite.

### RESULTS

### *Experiment A*

A solution of the metabolite in ethanol possessed a bright blue fluorescence which changed immediately to yellow on addition of sodium hydroxide. Absorption spectroscopy in hexane revealed the presence of absorbing impurity in the shorter wave length region but above 280 mμ the bands recorded (Fig. 1, Table I) were, with one exception (see DISCUSSION), almost identical with those of synthetic 2′-hydroxybenzanthracene.

TABLE I

Absorption bands in hexane (mμ)

| Rabbit faecal metabolite | 2′–OH | Mouse metabolite BA–OH | Methylated rabbit metabolite | 2′–OMe |
|---|---|---|---|---|
| — | 226–227 | 226 | 226–227 | 227 |
| — | [255–256] | 253 | [254–256] | [254–256] |
| — | 262 | [262] | 262–263 | 262–263 |
| — | 269–270 | [270–272] | 270 | 269 |
| — | [278–280] | [278–280] | 278–280 | 280 |
| 286 | 286 | 286 | 287 | 287 |
| 297 | 297 | 297 | 297 | 297·5 |
| 322–324 | 325·5 | [320–324] | 324–326 | 326–327 |
| 337 | — | 337 | — | — |
| — | 341 | [340–341] | 338–342 | 340–342 |
| 354 | 354 | 354–355 | 354 | 354·5 |
| 373 | 373 | 372 | 372 | 372 |
| 394 | 394 | 394 | 393 | 393 |

2′–OH = 2′–hydroxybenzanthracene.
2′–OMe = 2′-methoxybenzanthracene.
BA–OH = free phenolic component of the mouse sulphate conjugate.

Methylation of the metabolite with dimethyl sulphate and excess sodium hydroxide yielded a neutral derivative, purified by chromatography on alumina from benzene followed by alumina from cyclohexane. The absorption spectrum of this compound proved to be almost identical with that of 2′-methoxybenzanthracene (Fig. 2, Table I) and the phenolic derivative excreted in the faeces was therefore concluded to be 2′-hydroxybenzanthracene.

Also included for comparison in the above data (Fig. 1, Table I) is the absorption spectrum of the phenolic fraction obtained from the sulphate conjugate of mouse bile (Harper, 1959*a*). This again is very similar to that of the 2′-hydroxy derivative but differs from it in the sharpness of the major band at 286 mμ. In this respect it is similar to 4′-hydroxybenzanthracene and a probable explanation of this behaviour is that a mixture of the 2′- and 4′-hydroxy compounds is present, with the former in excess.

### *Experiment B*

The primary aim of this experiment, to isolate conjugated metabolites from the gall bladder and small intestine, was prevented by the large amount of acidic colouring matter present in these organs. Recourse was had therefore to the hydrolytic procedure described under MATERIALS AND METHODS in the hope

FIG. 1.—Absorption spectra in hexane.

1. Phenolic component of the mouse sulphate conjugate (*b* ordinates).
2. 2′-Hydroxy-1 : 2-benzanthracene (*b* ordinates).

3A and 3B.—Weak and concentrated solutions of the phenolic metabolite of benzanthracene from rabbit faeces (3A—*a* ordinates ; 3B—*b* ordinates).

FIG. 2.—Absorption spectra in hexane.

1. 2′-Methoxy-1 : 2-benzanthracene (*b* ordinates).
2. Methylated phenolic metabolite of benzanthracene from rabbit faeces (*a* ordinates).

that a phenolic derivative liberated by this means from a sulphate conjugate would be more amenable to isolation and purification. In this respect the experiment was a success and the phenolic fraction thus obtained, although impure, possessed absorption bands identical with those of the faecal metabolite.

### DISCUSSION

Although there can be little doubt that the rabbit metabolite isolated in this work is 2′-hydroxy-benzanthracene it is perhaps surprising, in view of the close correspondence existing between other bands, that the rather diffuse 341 m$\mu$ band of the synthetic compound should appear at 337 m$\mu$ in the metabolite. This divergence, however, is undoubtedly due to the presence of absorbing impurity for, as absorption in a mixture is additive, the superimposed effect of decreasing background absorption upon a broad, almost horizontal maximum of this nature will be to decrease absorption in the band passing from shorter to longer wave lengths. The overall effect therefore will be a sharpening and hypsochromic displacement of the maximum. Significantly, this effect was not observed with the purer methylated form of the metabolite.

The isolation of free 2′-hydroxybenzanthracene from the gall bladder and small intestine following acid hydrolysis is consistent with the presence in these organs of a sulphate conjugate, if not perhaps the glucuronide, of the phenol. The situation in respect therefore is probably similar to that occurring in the mouse but differs from it in the fact that hydroxylation occurs exclusively in the 2′-position. The metabolism of 1 : 2-benzanthracene in the mouse is indeed analogous to that of 3 : 4-benzpyrene for, although hydroxylation occurs at more than one point of the molecule, the quantitative relationship existing between the resultant products appears to be time dependent. Thus the initial major products yielded by these two hydrocarbons *in vivo* are probably 2′-hydroxy-benzanthracene and the unidentified $F_1$ metabolite of 3 : 4-benzpyrene but at later time intervals these are replaced by the structurally analogous 4′-hydroxy-benzanthracene and 8-hydroxybenzpyrene. It would appear therefore that the initial products of hydroxylation in the mouse are themselves inhibitors of their own synthesis and herein may lie the secret both of species differences in the site of hydroxylation and in the graded carcinogenic response elicited by the hydrocarbons in these species.

In conclusion it can be said that the hydroxylation of 1 : 2-benzanthracene in the 2′-position and the lack of carcinogenic activity in the rabbit is consistent with the proposal advanced previously (Harper, 1959*b*) that an ortho form of binding is favoured during hydroxylation in the microsomes of this species. The carcinogenic action of this hydrocarbon in the mouse is then to be envisaged as a secondary effect arising from an initial " poisoning " of the defensive ortho-mechanism which then allows the active para form to come into play. Similar considerations may also apply in the case of 1 : 2 : 5 : 6-dibenzanthracene (without activity in the rabbit) and 3 : 4-benzpyrene (reduced activity in the rabbit).

Data of carcinogenic activity are taken from Hartwell (1951).

### SUMMARY

1. 1 : 2-Benzanthracene has been found to undergo hydroxylation in the 2′-position after intraperitoneal and intravenous injection in the rabbit. The phenolic metabolite, 2′-hydroxybenzanthracene, is excreted in the faeces although it is probably eliminated initially via the bile in conjugated form.

2. Previous data obtained on the metabolism of 1 : 2-benzanthracene in mice have been re-examined and are consistent with the view that hydroxylation occurs in both the 2′- and 4′-positions with the former predominating during the initial stages of metabolism.

3. These results are discussed in relation to former proposals concerning hydroxylation and carcinogenesis.

I am greatly indebted to Dr. R. Schoental for her gift of 2′-methoxybenzanthracene and to Professor G. M. Badger for his gift of 2′- hydroxybenzanthracene.

The expenses of this work were defrayed from a block grant by the British Empire Cancer Campaign.

### REFERENCES

BERENBLUM, I. AND SCHOENTAL, R.—(1943) *Cancer Res.*, **3**, 686.

HARPER, K. H.—(1959*a*) *Brit. J. Cancer*, **13**, 718.—(1959*b*) *Ibid.*, **13**, 732.

HARTWELL, J. L.—(1951) ' Survey of Compounds which have been Tested for Carcinogenic Activity '. 2nd Ed. Bethesda (National Cancer Inst.).